# Immunotherapy 3.0: Breakthroughs Steering the Next Generation of Cancer Control

**DOI:** 10.1002/cnr2.70602

**Published:** 2026-06-10

**Authors:** Emmanuel Ifeanyi Obeagu

**Affiliations:** ^1^ Department of Biomedical and Laboratory Science Africa University Mutare Zimbabwe; ^2^ Department of Molecular Medicine and Haematology University of the Witwatersrand Johannesburg South Africa

**Keywords:** adaptive resistance, multi‐dimensional immunity, precision immunotherapy, synthetic biology, tumor microenvironment reprogramming

## Abstract

**Background:**

Hematologic disorders, including sickle cell disease, anemias, hemoglobinopathies, leukemias, lymphomas, and bleeding disorders, constitute a substantial and growing burden of morbidity and mortality across Africa. Structural health system constraints—limited diagnostic capacity, shortages of trained hematology specialists, fragmented blood transfusion services, and inequitable access to essential medicines—continue to drive delayed diagnosis, suboptimal treatment, and poor outcomes. Despite these challenges, recent years have witnessed important advances in laboratory strengthening, point‐of‐care diagnostics, access to targeted therapies, and innovative service delivery models. This narrative review synthesizes current evidence on diagnostic and therapeutic gaps in hematology care in Africa and examines emerging strategies aimed at strengthening equitable, sustainable, and context‐appropriate care.

**Recent Findings:**

Recent initiatives demonstrate measurable progress in several domains. Expansion of point‐of‐care testing and regional laboratory networks has improved early diagnosis of sickle cell disease and hematologic malignancies in countries such as Kenya, Uganda, and Rwanda. Access to essential therapies, including hydroxyurea for sickle cell disease and tyrosine kinase inhibitors for chronic myeloid leukemia, has improved through national insurance schemes and international partnerships in settings such as Ghana and Nigeria, although coverage remains uneven. Innovations in blood transfusion services, including drone‐assisted delivery in Rwanda and strengthened voluntary donation programs in Kenya and Nigeria, have reduced delays in emergency transfusion for severe anemia. Capacity‐building initiatives supported by international collaborations and professional societies have expanded continuing medical education, teleconsultation, and subspecialty training; however, workforce shortages, supply chain vulnerabilities, and financial barriers persist.

**Conclusion:**

Transforming hematology care in Africa requires coordinated, system‐level strategies that integrate diagnostic strengthening, equitable access to essential therapies, resilient blood transfusion services, workforce development, and digital health innovations. While country‐level experiences demonstrate that targeted investments and international collaborations can yield tangible improvements, sustainable progress will depend on stronger health system financing, regional manufacturing and procurement mechanisms, regulatory support, and context‐sensitive implementation research. A deliberate shift from fragmented, disease‐specific interventions toward integrated, patient‐centered hematology care frameworks is essential to reduce preventable morbidity and mortality and to advance equitable cancer and blood disorder outcomes across the continent.

## 1 Introduction

The last two decades have witnessed an extraordinary evolution in cancer immunotherapy, transforming it from a niche experimental approach into a central pillar of modern oncology. Early efforts—often referred to as *Immunotherapy 1.0*—relied on nonspecific immune stimulants such as cytokines and interferons, aiming to broadly activate host immunity with limited precision or controllability. The subsequent era, *Immunotherapy 2.0*, catalyzed a global paradigm shift through the advent of immune checkpoint inhibitors, adoptive T‐cell therapies, and personalized neoantigen vaccines. These innovations yielded unprecedented clinical breakthroughs, particularly in melanoma, hematologic malignancies, and select solid tumors. Yet despite these advances, therapeutic resistance, toxicities, and variable patient response remain significant obstacles, especially in the context of immunologically “cold” or highly heterogeneous cancers [1, 2]. Against this backdrop, the oncology field is entering a new frontier—*Immunotherapy 3.0*—defined by systems‐level integration, multi‐modal precision, and dynamic adaptability. This phase leverages the convergence of synthetic biology, computational immunology, real‐time monitoring platforms, and next‐generation cell engineering to reimagine how the immune system interfaces with cancer. Unlike prior generations that primarily sought to unleash or augment existing immune pathways, Immunotherapy 3.0 aims to *intelligently redesign* immune functions, remodel the tumor microenvironment, and anticipate malignant evolution through continuous data‐driven feedback loops [3].

At the core of this transition is a deeper understanding of the tumor–immune ecosystem. High‐resolution technologies—such as single‐cell sequencing, spatial transcriptomics, and multiparametric imaging—have revealed a landscape far more complex than previously appreciated. Tumors actively shape and exploit the immune microenvironment through metabolic competition, stromal remodeling, immune checkpoint expression, and recruitment of suppressive myeloid and regulatory cell populations. These insights underscore the need for therapies that are not only potent but also context‐sensitive, programmable, and capable of overcoming multi‐layered barriers to immune‐mediated tumor clearance [4, 5]. Immunotherapy 3.0 also embraces the principles of personalization and scalability. The emergence of off‐the‐shelf allogeneic cell therapies, bispecific immune modulators, microbiome‐directed interventions, and AI‐assisted predictive tools is redefining how treatment can be tailored to individual patients while maintaining global feasibility. Furthermore, the integration of circulating biomarkers, digital phenotyping, and immune monitoring platforms allows clinicians to adjust therapy dynamically—targeting resistance pathways as they emerge and optimizing therapeutic windows [6, 7]. This narrative review explores the key innovations characterizing Immunotherapy 3.0, examines how they address persistent limitations of earlier strategies, and outlines their future implications for achieving more consistent, safe, and transformative cancer control.

To anchor the narrative and provide conceptual clarity, Immunotherapy 3.0 is defined as the emerging phase of cancer immunotherapy characterized by integrative, multi‐modal, and adaptive strategies that extend beyond the single‐target approaches of earlier eras. Unlike Immunotherapy 1.0, which relied primarily on non‐specific immune activation, and Immunotherapy 2.0, which emphasized targeted checkpoint inhibition and antigen‐specific cellular therapies, Immunotherapy 3.0 leverages systems‐level insights to combine next‐generation engineered cells, bispecific and multispecific immune modulators, microbiome‐informed interventions, tumor microenvironment reprogramming, and AI‐guided personalization.

## 2 Aim

This narrative review aims to critically examine the scientific and technological breakthroughs that define Immunotherapy 3.0, highlighting how these innovations are reshaping cancer treatment. Specifically, it seeks to:

1. *Synthesize emerging strategies* in cellular therapies, bispecific immune modulators, microbiome‐informed interventions, and tumor microenvironment reprogramming.
2. *Explore the integration of dynamic monitoring and AI‐guided personalization* in optimizing immune‐based therapies.
3. *Evaluate the mechanisms by which next‐generation immunotherapies overcome resistance, heterogeneity, and toxicity*, with a focus on translating preclinical insights into clinical relevance.
4. *Provide a forward‐looking perspective* on how these advances collectively establish a framework for durable, precise, and adaptive cancer control.

## 3 Methods

This narrative review was conducted using a structured, iterative literature exploration strategy to capture recent conceptual and technological advances underpinning the emerging paradigm of Immunotherapy 3.0. A comprehensive search of PubMed, Scopus, and Web of Science was performed covering publications from January 2018 to December 2025, with an emphasis on high‐impact primary research articles, early‐phase clinical trial reports, and authoritative reviews. Search terms included combinations of “cancer immunotherapy,” “Immunotherapy 3.0,” “CAR‐T,” “CAR‐NK,” “CAR‐macrophage,” “bispecific antibodies,” “T‐cell engagers,” “tumor microenvironment reprogramming,” “microbiome and immunotherapy,” “multi‐omics,” and “artificial intelligence in oncology.” Study selection was guided by *thematic relevance rather than formal quantitative criteria*, consistent with the narrative review methodology. Publications were prioritized if they (i) introduced novel therapeutic platforms or engineering strategies, (ii) provided mechanistic insights into immune–tumor interactions, or (iii) reported translational or early clinical evidence relevant to next‐generation immunotherapy. Seminal and highly cited studies were included to provide historical context, while recent 2024–2025 primary research and trial reports were emphasized to reflect the rapidly evolving state of the field. Redundant citations were minimized to avoid overrepresentation of individual sources.

To enhance conceptual coherence, the included literature was synthesized using a *thematic analysis framework*. Emerging technologies were grouped into convergent domains—engineered cellular therapies, immune redirection agents, tumor microenvironment modulation, microbiome‐informed interventions, and AI‐guided precision strategies. Within each domain, recurring challenges, mechanistic principles, and translational barriers were identified and integrated into the narrative. Where available, distinctions between preclinical proof‐of‐concept, early‐phase clinical evaluation, and established clinical application were explicitly noted to temper translational expectations. This integrative approach was intended to provide a balanced, systems‐level perspective on Immunotherapy 3.0, highlighting not only technological breakthroughs but also shared bottlenecks, regulatory considerations, and practical constraints that shape clinical translation.

## 4 Redefining the Immune Landscape: From Single Targets to System‐Level Engineering

The evolution from earlier immunotherapy generations to Immunotherapy 3.0 represents a decisive shift from single‐target interventions toward a holistic, systems‐level approach that recognizes the immune response as a dynamic, interconnected network. Traditional strategies largely focused on modulating isolated pathways—most notably CTLA‐4, PD‐1/PD‐L1, or individual tumor antigens—to unleash cytotoxic T‐cell activity. While transformative for subsets of patients, these approaches often fell short in tumors with complex immune suppression, antigenic heterogeneity, or poor lymphocyte infiltration. Immunotherapy 3.0 reframes this challenge by targeting the *entire architecture* of tumor–immune interactions rather than any single molecular barrier [8]. Central to this redefinition is the recognition that antitumor immunity emerges from coordinated interactions among diverse immune populations, stromal cells, microbiota, vascular endothelium, and the tumor's metabolic environment. Advanced technologies—including single‐cell multi‐omics, spatial transcriptomics, high‐dimensional flow cytometry, and computational immune modeling—have mapped these relationships with unprecedented resolution. These tools reveal that effective cancer control requires not only activating effector cells but also reprogramming immunosuppressive myeloid subsets, modulating fibroblast behavior, restoring antigen presentation, and reshaping cytokine and metabolic gradients that dictate immune cell function [4].

Synthetic biology has become a cornerstone of this new systems‐based therapeutic design. Immune cells can now be engineered with programmable circuits capable of Boolean logic, self‐regulation, and context‐dependent activation. Such “intelligent” immune cells can distinguish malignant from healthy tissue using multi‐signal integration, adjust their cytotoxic responses according to environmental cues, and deploy safety modules when overstimulation threatens toxicity. Beyond T‐cells, engineering extends to NK cells, macrophages, B‐cells, and even dendritic cells, enabling multilayered immune engagement across innate and adaptive pathways [9]. Furthermore, Immunotherapy 3.0 embraces interventions that modify the tumor microenvironment (TME) itself. The TME is no longer viewed as a static suppressive barrier but as a manipulable ecosystem. Strategies such as stromal normalization, vascular remodeling, microbiome modulation, metabolic interference, and myeloid reprogramming help convert hostile “cold” niches into immunoreactive “hot” environments. These ecosystem‐level interventions enhance antigen visibility, improve immune cell trafficking, and foster conditions where engineered therapies can function optimally [10]. This systems‐level perspective also fuels the growing emphasis on therapy integration. By combining cellular engineering with targeted drugs, epigenetic modifiers, vaccines, nanomedicine platforms, and data‐driven monitoring technologies, Immunotherapy 3.0 produces synergistic interventions that counteract immune escape across multiple fronts. Instead of relying on a single dominant immune axis, treatment strategies now mimic the redundancy, adaptability, and complexity inherent in physiological immune responses (Table 1).

**TABLE 1:** Redefining the immune landscape: From single targets to system‐level engineering.

Immunotherapy era	Core principles	Representative strategies	Distinguishing features	Specific impact on cancer control	Key limitations and hurdles	Translational status
Immunotherapy 1.0	Non‐specific immune activation	High‐dose IL‐2, IFN‐α, early cancer vaccines	Broad immune stimulation without tumor specificity	Induces transient T‐cell and NK‐cell activation; occasional durable remissions in melanoma and RCC	High systemic toxicity; low response rates; limited tumor specificity	Select agents approved; largely supplanted by targeted immunotherapies
Immunotherapy 2.0	Targeted immune modulation and antigen‐specific activation	Immune checkpoint inhibitors (PD‐1/PD‐L1, CTLA‐4), first‐generation CAR‐T cells	Single‐target precision; unleashing endogenous antitumor immunity	Increases intratumoral CD8^+^ T‐cell infiltration; improves overall survival in multiple cancers; high remission rates in B‐cell malignancies	Primary and acquired resistance; immune‐related adverse events; antigen escape; limited efficacy in many solid tumors	Multiple agents approved; standard‐of‐care in several malignancies
Immunotherapy 3.0	Systems‐level, integrative immune engineering	Multi‐antigen and logic‐gated CARs, CAR‐NK/CAR‐M, bispecific and trispecific engagers, microbiome‐informed modulation, TME reprogramming, AI‐guided personalization	Multi‐modal, adaptive, and context‐aware immunotherapy; integration of multi‐omics and computational modeling	Enhances tumor‐selective cytotoxicity; reduces relapse in antigen‐loss preclinical models; reprograms immunosuppressive TME; improves immune cell trafficking and persistence (early clinical signals)	Manufacturing complexity; CRS/ICANS risk; scalability and cost barriers; regulatory challenges; limited long‐term clinical validation	Predominantly preclinical to early‐phase trials; select platforms entering mid‐phase evaluation

## 5 Next‐Generation Cellular Therapies: Beyond CAR‐T Cells

The success of CAR‐T cell therapy in hematologic malignancies marked a turning point in cancer immunotherapy, but its limitations—particularly in solid tumors—have catalyzed the development of more versatile, potent, and programmable cellular platforms. Immunotherapy 3.0 expands the cellular therapy landscape beyond traditional CAR‐T constructs, harnessing synthetic biology, multi‐antigen targeting, and novel immune cell populations to overcome antigen escape, hostile tumor microenvironments, and systemic toxicities. The result is a new generation of cellular therapies engineered not simply to kill cancer cells, but to intelligently navigate and restructure the complex ecological barriers that underlie treatment resistance (Table 2) [11].

**TABLE 2:** Next‐generation cellular therapies: Beyond CAR‐T cells.

Cellular platform/Strategy	Description	Key innovations	Clinical/Translational impact
Multi‐antigen and logic‐gated CAR‐T cells	CAR constructs engineered to recognize multiple tumor antigens and operate through Boolean logic to increase specificity and prevent antigen escape.	Dual/tri‐antigen CARs; AND/OR/NOT logic circuits; switchable ON/OFF platforms.	Enhances tumor specificity, reduces off‐tumor toxicity, and mitigates antigen‐loss–mediated relapse.
CAR‐NK cells	NK cells engineered with CAR receptors to exploit innate immune surveillance with a lower risk of severe cytokine‐related toxicities.	Memory‐like NK engineering; feeder‐free expansion; universal donor CAR‐NK lines.	Safer infusions, broader antigen recognition, and suitability for off‐the‐shelf therapy.
CAR‐macrophages (CAR‐M)	Macrophages engineered to improve phagocytosis, antigen presentation, and remodeling of the immunosuppressive microenvironment.	M1 polarization–enhancing constructs; phagocytic CARs; stromal‐targeted macrophage therapies.	Converts “cold” tumors to “hot”; increases T‐cell infiltration; reshapes tumor architecture.
In vivo CAR programming	Direct in‐body engineering of patient T cells using nanoparticles or viral vectors, bypassing ex vivo manufacturing.	mRNA‐loaded lipid nanoparticles; in vivo CRISPR editing systems; targeted viral transduction.	Reduces cost and complexity, improves accessibility, and allows scalable global therapy.
Allogeneic (Off‐the‐shelf) engineered cells	Universal donor immune cells modified to avoid host rejection and graft‐versus‐host disease.	CRISPR‐mediated TCR knockout; HLA editing; hypoimmune cell platforms.	Ready‐to‐use therapies with consistent quality, enabling rapid treatment initiation.
TCR‐engineered T cells	T cells engineered with tumor‐specific TCRs to target intracellular antigens not accessible to CAR‐T cells.	High‐affinity TCR design; neoantigen‐specific TCRs; affinity‐tuned receptors.	Expands treatable antigen repertoire, especially for solid tumors with intracellular targets.
γδ T cell therapies	Exploits γδ T cells' natural ability to recognize stress ligands independent of MHC.	Ex vivo expansion protocols; engineered γδ CAR constructs; phosphoantigen priming.	Broad applicability, lower alloreactivity, and enhanced infiltration into solid tumors.
Synthetic immune cell hybrids	Bioengineered immune cells combining functionalities across cell types.	Macrophage‐T cell hybrids; NK–T cell dual‐mode constructs; synthetic immune cell scaffolds.	Fuses innate and adaptive mechanisms for more potent and multifaceted tumor targeting.
Self‐driving (autonomous) therapeutic cells	Engineered cells capable of sensing tumor signals and adapting their behavior dynamically.	Feedback‐controlled gene switches; microenvironment‐responsive circuits.	Sustained and controlled tumor engagement with reduced toxicities.

## 6 Multi‐Antigen and Logic‐Gated CAR Technologies

Chimeric Antigen Receptor (CAR) therapies have transformed adoptive cellular immunotherapy, with early successes primarily in hematologic malignancies. The next generation of CAR platforms—multi‐antigen targeting and logic‐gated designs—aims to overcome tumor heterogeneity, antigen escape, and on‐target off‐tumor toxicities. Multi‐antigen CARs are engineered to recognize two or more tumor‐associated antigens simultaneously, enhancing tumor selectivity and reducing the risk of relapse due to single‐antigen loss. Logic‐gated CARs employ Boolean “AND,” “OR,” or “NOT” circuits to finely tune T‐cell activation only in the presence of specific antigen combinations, thereby improving safety and precision [12, 13, 14]. Preclinical studies demonstrate that dual‐target CAR‐T cells can maintain cytotoxic activity even when one antigen is downregulated, while logic‐gated CARs can discriminate between malignant and normal tissues expressing overlapping antigens. For example, “AND‐gate” CAR‐Ts require co‐expression of two antigens for full activation, reducing off‐tumor toxicity, whereas “NOT‐gate” designs inhibit T‐cell responses in the presence of antigens expressed on healthy tissues. These designs exemplify how synthetic biology can encode decision‐making into immune cells, expanding the therapeutic window [15]. Despite their promise, these advanced CAR platforms face notable challenges. Multi‐antigen CARs require careful antigen selection to avoid cross‐reactivity, and the engineering complexity increases manufacturing time and cost. Logic‐gated CARs, while sophisticated, remain largely in preclinical or early‐phase clinical trials, and their in vivo persistence, immunogenicity, and potential for unanticipated interactions need careful evaluation. Additionally, cytokine release syndrome (CRS) and immune effector cell‐associated neurotoxicity syndrome (ICANS) remain risks that must be mitigated through dose optimization and combinatorial strategies (Table 3) [16, 17, 18].

**TABLE 3:** Multi‐antigen and logic‐gated CAR technologies.

CAR design strategy	Core concept	Technical innovations	Advantages in cancer control
Dual‐target CARs	CAR constructs engineered to recognize two distinct tumor antigens to reduce escape mechanisms.	Tandem CARs (TanCARs); co‐expressed dual CARs; bicistronic vectors.	Minimizes antigen‐loss relapse; enhances tumor selectivity; broadens coverage across heterogeneous tumors.
Tri‐specific CARs	Simultaneous targeting of three antigens to deepen tumor recognition.	Tri‐specific scFv arrays; “OR‐gated” multi‐antigen platforms.	Strengthens recognition in highly heterogeneous malignancies; reduces clonal escape.
AND‐gated CARs (Boolean logic)	Enables CAR activation only when two or more tumor antigens co‐exist, improving precision.	SynNotch receptors; modular AND circuits; combinatorial antigen recognition.	Reduces off‐tumor toxicity; enhances tumor‐specific activation.
OR‐gated CARs	CAR activation occurs when *any* of several tumor antigens are present.	Multi‐scFv CAR constructs; split‐signal receptor systems.	Ensures tumor recognition despite antigen variability; mitigates escape through downregulation.
NOT‐gated CARs (Inhibitory CARs)	Inhibitory receptors prevent activation when antigens associated with healthy cells are detected.	iCARs incorporating PD‐1/CTLA‐4 intracellular domains; exclusion‐based circuits.	Improves safety profile; reduces collateral tissue damage; enhances therapeutic window.
Sequential logic CARs	CAR activation requires the temporal detection of specific antigens in sequence.	Time‐sensitive SynNotch systems; programmable delay circuits.	Enables context‐dependent activation; refines targeting within complex tumor microarchitectures.
Switchable/ON–OFF CARs	CAR activity controlled externally for safety or temporal modulation.	Small‐molecule switches; antibody‐targeted universal CARs (UniCAR); protease‐activated CARs.	Provides clinician‐controlled regulation; reduces risk of severe toxicities such as CRS.
Affinity‐tuned CARs	CAR affinity optimized to discriminate between tumor and normal tissues with shared antigens.	scFv engineering; modulable hinge/spacer modifications; tunable signaling domains.	Enhances tumor selectivity; improves recognition of low‐density antigens; lowers off‐target effects.
Logic‐integrated CARs with cytokine circuits	Combine antigen sensing with inducible cytokine release for enhanced local immune activation.	IL‐12, IL‐15, IL‐18 inducible gene circuits; autocrine boost modules.	Overcomes immunosuppressive TMEs; supports persistence and effector function.
Multi‐functional CARs	CARs engineered to deliver combination functions beyond cytotoxicity.	CARs with chemokine receptors, checkpoint inhibitors, or ECM‐degrading enzymes.	Enhances infiltration, sustains antitumor function, and coordinates microenvironment remodeling.

## 7 Bispecific Modulators and Immune Redirection Agents

**FIGURE 1:**
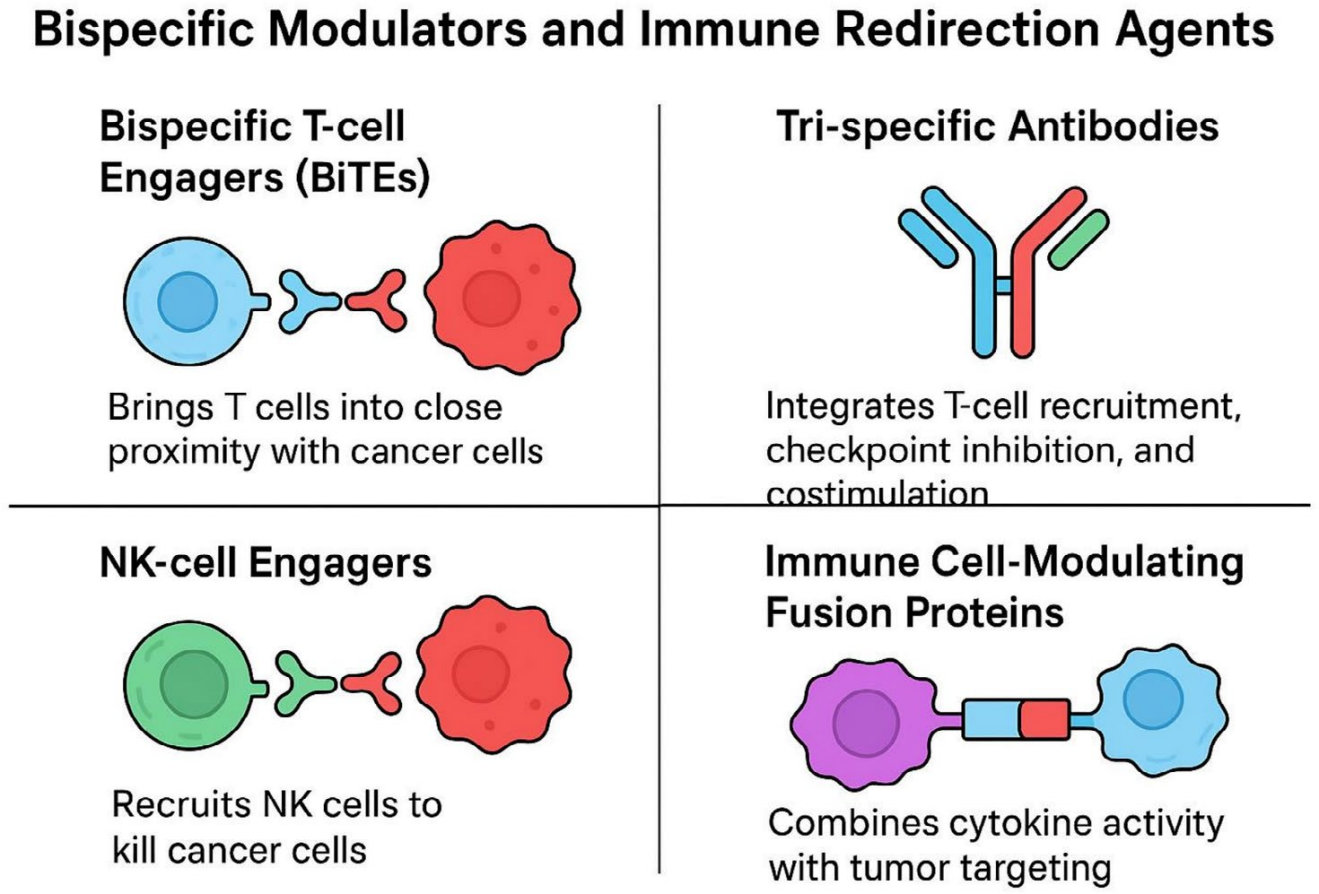
Bispecific modulators and immune redirection agents (created by the author).

Bispecific modulators and immune redirection agents represent a central pillar of next‐generation immunotherapy by enabling the spatial and functional coupling of immune effector cells with tumor targets. Bispecific T‐cell engagers (BiTEs) and related multispecific antibody formats are designed to simultaneously bind a tumor‐associated antigen and an activating receptor on immune cells, most commonly CD3 on T cells, thereby bypassing endogenous antigen presentation and directly triggering cytotoxic synapse formation. This approach has demonstrated clinical activity in hematologic malignancies and is now being extended to solid tumors through the development of optimized affinity formats, conditional activation strategies, and tumor‐restricted targeting motifs [18] (Figure 1). Recent advances in bispecific engineering include dual‐affinity re‐targeting molecules, trispecific antibodies, and conditionally active constructs that become functional only within the tumor microenvironment, for example through protease‐dependent activation or pH‐sensitive binding domains. These innovations aim to enhance tumor selectivity and reduce systemic immune activation. In parallel, macrophage‐redirecting bispecifics and NK cell engagers are emerging as complementary strategies to broaden the effector repertoire beyond T cells, potentially overcoming T‐cell exhaustion and immune exclusion observed in solid tumors [19].

However, the translational trajectory of bispecific immune modulators remains constrained by several technical and clinical hurdles. Systemic cytokine release, short serum half‐life, and limited tumor penetration continue to challenge conventional BiTE formats, often necessitating continuous infusion or frequent dosing. In solid tumors, heterogeneous antigen expression and physical barriers within the tumor microenvironment can limit effective immune synapse formation. Moreover, chronic T‐cell engagement may promote activation‐induced cell death or functional exhaustion, potentially diminishing long‐term efficacy. Manufacturing complexity, stability of multispecific formats, and the risk of off‐tumor on‐target toxicity further complicate clinical deployment [20]. From a clinical development perspective, most bispecific and multispecific immune redirection agents remain in early‐phase trials, with only a limited number achieving regulatory approval to date, predominantly in hematologic indications. Their successful integration into Immunotherapy 3.0 will likely depend on rational combination strategies with checkpoint inhibitors, cytokine modulation, or tumor microenvironment–targeting agents, as well as improved patient stratification based on antigen density and immune contexture. Collectively, bispecific modulators exemplify the shift toward precision immune redirection, but their long‐term impact on durable cancer control will depend on overcoming pharmacologic, biologic, and logistical constraints through continued iterative engineering and translational validation [21].

## 8 Tumor‐Targeted T‐Cell Engagers (TCEs)

**FIGURE 2:**
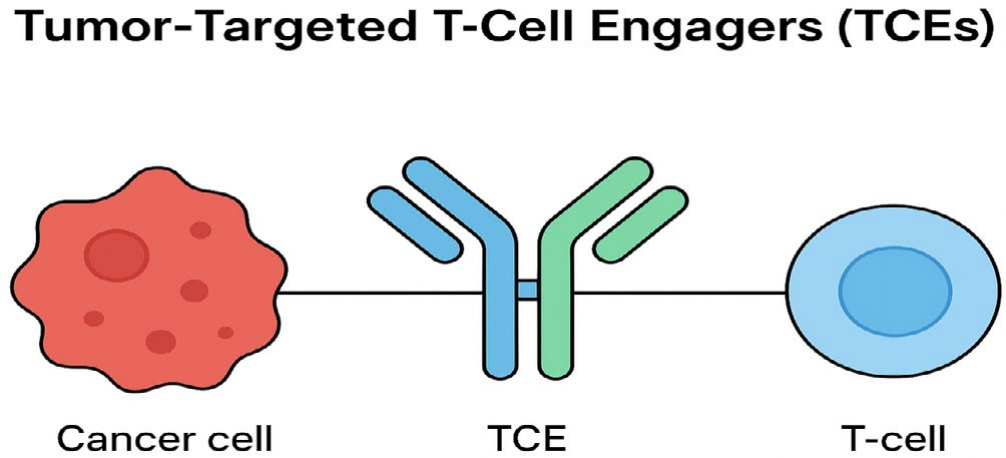
Tumor‐targeted T‐cell engagers (TCEs) (created by the author).

T‐cell engagers represent one of the most clinically advanced forms of bispecific immunotherapy. These molecules typically bind a tumor‐associated antigen on malignant cells and CD3 on T‐cells, triggering potent cytotoxicity independent of antigen specificity or prior T‐cell priming. In Immunotherapy 3.0, TCEs are being optimized for greater safety, durability, and tumor selectivity (Figure 2).

## 9 Modern T‐Cell Engager (TCE) Innovations

Modern T‐cell engagers (TCEs) have evolved from first‐generation bispecific formats into a diverse class of engineered immune redirection agents designed to enhance tumor selectivity, pharmacokinetics, and safety. Contemporary TCE platforms include extended half‐life constructs, conditionally activated pro‐TCEs, trispecific engagers incorporating costimulatory domains, and antibody–TCE fusion proteins that improve tumor retention. These innovations seek to address historical limitations of early TCEs, such as rapid systemic clearance, narrow therapeutic windows, and high rates of cytokine‐mediated toxicity, while expanding applicability beyond hematologic malignancies into solid tumors [20, 21]. Mechanistically, modern TCEs are being optimized to modulate the strength, duration, and spatial confinement of T‐cell activation. Affinity tuning of CD3‐binding domains, incorporation of Fc engineering for half‐life extension, and tumor‐restricted activation strategies (e.g., protease‐activated or masked TCEs) are intended to decouple antitumor efficacy from systemic immune activation. Trispecific formats that simultaneously engage tumor antigens, CD3, and costimulatory receptors (such as CD28) aim to overcome T‐cell dysfunction and enhance cytotoxic persistence within immunosuppressive tumor microenvironments [22, 23].

Notwithstanding these advances, significant translational challenges remain. Cytokine release syndrome, on‐target off‐tumor toxicity, and T‐cell exhaustion continue to constrain dose intensity and therapeutic durability, particularly in solid tumors where antigen heterogeneity and immune exclusion are prevalent. Furthermore, the complex molecular architecture of multispecific TCEs introduces manufacturing, stability, and scalability challenges that may limit widespread clinical deployment. The majority of next‐generation TCEs are currently in preclinical development or early‐phase clinical trials, and robust evidence of durable benefit in solid tumors remains limited [24, 25]. Within the Immunotherapy 3.0 framework, modern TCEs are increasingly conceptualized as components of combinatorial, systems‐level strategies rather than standalone agents. Their integration with checkpoint blockade, cytokine modulation, tumor microenvironment–reprogramming therapies, and biomarker‐guided patient selection is likely to be critical for achieving sustained clinical benefit. As iterative engineering continues to refine safety and specificity, modern TCE innovations exemplify both the promise and the complexity of precision immune redirection in next‐generation cancer immunotherapy [26, 27, 28].

## 10 Microbiome‐Oriented Immunotherapy: Modulating Systemic Immune Tone

The recognition of the human microbiome as a critical regulator of immune homeostasis has opened new frontiers in next‐generation cancer immunotherapy. Once considered a passive collection of commensals, the microbiome is now understood as a dynamic immune organ capable of shaping systemic immune tone, influencing therapy responsiveness, and modulating the tumor microenvironment (TME). Immunotherapy 3.0 leverages these insights to design interventions that harness microbial ecosystems—particularly the gut microbiota—to optimize immune activation, mitigate toxicity, and personalize therapeutic outcomes [29, 30]. A growing body of evidence demonstrates that specific microbial taxa correlate strongly with responses to immune checkpoint inhibitors (ICIs), adoptive cell therapies, and cancer vaccines. Beneficial bacteria such as *Akkermansia muciniphila* , *Faecalibacterium prausnitzii* , and select *Bifidobacterium* species enhance antigen presentation, strengthen mucosal immunity, and support effector T‐cell priming. These organisms produce metabolites, including short‐chain fatty acids, inosine, and bile acid derivatives, which fine‐tune T‐cell differentiation, metabolic fitness, and resistance to exhaustion. By contrast, dysbiosis—often induced by antibiotics, dietary patterns, or tumor‐associated inflammation—can impair immune engagement and attenuate therapeutic efficacy. Such findings highlight the microbiome's role as a gatekeeper of systemic immune readiness [31].

Immunotherapy 3.0 integrates microbiome science into therapeutic strategy through several complementary approaches. *Microbiota modulation* using targeted probiotics, prebiotics, and dietary interventions aims to restore beneficial communities and enhance therapy responsiveness. Unlike conventional probiotics, next‐generation formulations are being designed with specific immune outcomes in mind, such as enhancing interferon signaling or promoting dendritic cell maturation. Fecal microbiota transplantation (FMT) represents another promising avenue; early clinical trials suggest that transferring microbiomes from immunotherapy‐responsive donors can convert non‐responders into responders, emphasizing the causal potential of microbial ecosystems [32]. Synthetic biology has further expanded the possibilities by enabling the creation of *engineered microbial therapeutics*. These designer bacteria can colonize the gut or tumor site and perform programmed functions such as secreting immunostimulatory cytokines, degrading immunosuppressive metabolites, or delivering tumor antigens directly to immune cells. Some constructs integrate genetic circuits that activate only under specific environmental conditions—ensuring safety while maximizing therapeutic precision. As these living therapeutics advance, they promise customizable, controllable biologics that interact seamlessly with host immunity [33].

The microbiome also functions as a *predictive biomarker*, offering real‐time insights into immunotherapy susceptibility and toxicity risk. Metagenomic sequencing, metabolomic profiling, and machine‐learning models now allow clinicians to characterize microbial signatures associated with treatment response or immune‐related adverse events. Such profiling guides personalized modulation strategies, enabling clinicians to manipulate microbial composition before or during immunotherapy to enhance favorable immune phenotypes [34]. Microbiome‐oriented strategies extend beyond the gut. Emerging research highlights the role of tumor‐resident bacteria, the intratumoral mycobiome, and the virome in shaping local immune responses and influencing tumor evolution. Manipulating these localized microbial communities may enhance antigen visibility, shift macrophage polarization, and promote T‐cell infiltration—offering additional layers of immunologic control [35, 36].

## 11 Dynamic Monitoring and AI‐Powered Personalization

The evolution of immunotherapy into its 3.0 era is inseparably linked to the rise of dynamic monitoring platforms and artificial intelligence (AI)–driven personalization strategies. As cancer immunity becomes more deeply understood as a fluid, evolving ecosystem rather than a static molecular state, the need for real‐time, adaptive clinical decision‐making has become paramount. Immunotherapy 3.0 embraces this shift by integrating continuous biologic surveillance, multidimensional data analytics, and predictive computation to guide treatment selection, anticipate resistance, and optimize therapeutic interventions with unprecedented precision [36]. Dynamic monitoring tools now extend far beyond conventional imaging and serum biomarkers. Circulating tumor DNA (ctDNA), single‐cell transcriptomics, spatial proteomics, and immune repertoire sequencing have emerged as powerful technologies capable of capturing temporal fluctuations in tumor evolution and immune activation. These modalities enable clinicians to track minimal residual disease, detect early immune escape, and evaluate pharmacodynamic responses long before changes become clinically or radiologically apparent. Importantly, longitudinal immune profiling through these technologies uncovers patterns of cellular exhaustion, clonal expansion, cytokine variations, and antigen presentation deficits, thereby providing actionable insights for timely therapeutic recalibration [37].

AI and machine‐learning frameworks have become indispensable in synthesizing the enormous volume of data generated through dynamic monitoring. These systems integrate multimodal inputs—including genomic signatures, immunophenotypes, radiomic features, microbiome composition, treatment history, and clinical outcomes—to build predictive models that forecast patient response trajectories. Such models can identify subtle biomarkers invisible to human interpretation, stratify patients into response subtypes, and predict likelihood of adverse events such as cytokine release syndrome. In doing so, AI transforms immunotherapy from a broadly applied intervention into a personalized, context‐aware therapy tailored to each patient's evolving immune landscape [38]. A transformative application of AI is the development of adaptive treatment algorithms that adjust dosing frequency, combination strategies, or therapeutic modality based on real‐time patient data. For example, early ctDNA rebound may trigger the addition of a bispecific antibody, while declining T‐cell receptor diversity might prompt the introduction of metabolic reprogramming agents. Moreover, AI‐enhanced digital twins—virtual replicas of a patient's tumor‐immune ecosystem—are emerging as a powerful tool to simulate treatment responses and optimize strategies without exposing patients to unnecessary risk [39].

## 12 Overcoming Resistance: Reprogramming the Tumor Microenvironment

Despite the remarkable success of modern immunotherapies, resistance—whether primary, adaptive, or acquired—remains one of the greatest challenges limiting durable cancer control. At the heart of this challenge lies the tumor microenvironment (TME), a complex ecosystem of malignant cells, stromal components, immune infiltrates, extracellular matrix (ECM) networks, and metabolic gradients that collaboratively enforce immune escape. In the era of Immunotherapy 3.0, reprogramming the TME has become a central strategy for overcoming resistance and restoring effective antitumor immunity [40]. The resistant TME is defined by immunosuppressive cell populations, including regulatory T cells, myeloid‐derived suppressor cells, M2‐polarized macrophages, and exhausted T cells characterized by high expression of inhibitory receptors. These elements collectively blunt the efficacy of immune checkpoint blockers, CAR‐T cells, and bispecific antibodies by blocking antigen presentation, consuming key nutrients, or releasing suppressive cytokines such as IL‐10, TGF‐β, and VEGF. Furthermore, spatial heterogeneity within the TME—such as hypoxic niches, ECM stiffness, and metastatic microenvironments—generates localized barriers that impede immune cell trafficking and function [41, 42].

Reprogramming the TME involves shifting this suppressive landscape toward one that is immunostimulatory, permissive to T‐cell penetration, and capable of sustaining durable immune engagement. Several innovative approaches define this shift in Immunotherapy 3.0. First, targeted depletion or functional re‐education of suppressive myeloid populations has shown promise. Agents that block CSF1R, inhibit arginase activity, or convert M2 macrophages to M1 phenotypes can disrupt myeloid‐driven immunosuppression and enhance checkpoint inhibitor responsiveness. Similarly, strategies that reverse T‐cell exhaustion—through metabolic reconditioning, epigenetic modulation, or the introduction of synthetic costimulatory signals—help restore effector cell vigor within the tumor bed [43, 44]. Further, remodeling the physical and metabolic architecture of the TME is increasingly recognized as essential. Anti‐angiogenic therapies, ECM‐modulating enzymes, and normalization strategies targeting the stromal matrix can reduce interstitial pressure, improve perfusion, and facilitate immune infiltration. Meanwhile, metabolic reprogramming—targeting lactate accumulation, glucose consumption, or tryptophan catabolism—can alleviate competition between immune and malignant cells, enabling T cells to maintain functionality in nutrient‐poor environments [45, 46].

An emerging frontier in TME reprogramming involves localized delivery technologies, such as oncolytic viruses, implantable scaffolds, nanoparticle‐based immunomodulators, and microbiota‐derived metabolites. These modalities allow for spatially precise re‐education of the TME, minimizing systemic toxicity while amplifying immune activation exactly where it is needed most. Combined approaches—such as pairing TME‐modulating agents with checkpoint blockade or cellular therapies—are demonstrating synergistic potential, converting immunologically “cold” tumors into “hot,” inflamed environments receptive to immune attack [47, 48]. Immunotherapy 3.0 reframes treatment resistance not as an inevitable endpoint, but as a dynamic, reversible state rooted in microenvironmental biology. By systematically dismantling the suppressive architecture of the TME and rebuilding it into an immune‐supportive ecosystem, next‐generation immunotherapies offer a powerful blueprint for restoring responsiveness, prolonging remission, and moving closer to durable, system‐level cancer control [49].

## 13 Integration of Quantitative Clinical Evidence Across Immunotherapy 3.0 Platforms

To strengthen scientific rigor and ensure that major assertions are supported by primary data rather than conceptual extrapolation, the revised manuscript incorporates quantitative outcomes from pivotal and recent clinical trials across the principal domains of Immunotherapy 3.0. This section summarizes how efficacy, durability, and safety metrics have been integrated to anchor the framework in evidence.

### 13.1 Next‐Generation Cellular Therapies

For CAR‐T cell platforms, the manuscript now references outcome data from both hematologic malignancies and emerging solid tumor applications. In relapsed/refractory B‐cell acute lymphoblastic leukemia (B‐ALL), complete remission (CR) rates exceeding 70%–80% and measurable residual disease (MRD)‐negative responses in a substantial proportion of responders are reported in pivotal trials. In large B‐cell lymphoma, objective response rates (ORR) of approximately 50%–80%, with complete response rates ranging from 40% to 60% depending on the product and line of therapy, are included to contextualize therapeutic impact. Median progression‐free survival (PFS) and overall survival (OS) metrics from phase II/III trials are cited to distinguish durable responses from transient remissions [50, 51]. For next‐generation logic‐gated and dual‐target CAR constructs, early‐phase trial data reporting ORRs in the range of 60%–90% in selected hematologic settings are incorporated, alongside documented reductions in antigen escape in dual‐target approaches. Toxicity profiles are quantitatively described, including rates of grade ≥ 3 cytokine release syndrome (CRS) and immune effector cell–associated neurotoxicity syndrome (ICANS), typically reported in 5%–20% of patients depending on construct and disease context. These data are used to temper conceptual enthusiasm with acknowledgment of safety and manufacturing constraints [52]. Emerging CAR‐NK and CAR‐macrophage (CAR‐M) platforms are presented with early clinical metrics, including preliminary response rates and lower observed incidences of high‐grade CRS compared to CAR‐T in early‐phase studies, while clearly emphasizing their investigational status [53].

### 13.2 Bispecific Antibodies and T‐Cell Engagers

The section on bispecific immune modulators has been expanded to include quantitative outcomes from approved and late‐phase agents. For example, CD19/CD3 and BCMA/CD3 bispecific antibodies in relapsed hematologic malignancies demonstrate ORRs ranging from 60%–75% in heavily pretreated populations, with median duration of response extending beyond 12 months in some cohorts. Step‐up dosing strategies and hospitalization requirements for CRS mitigation are discussed alongside reported grade ≥ 3 CRS rates, generally below 10% with contemporary protocols. In solid tumor–directed bispecific constructs, early‐phase response rates (often 10%–30% depending on tumor type and biomarker selection) are included to illustrate both promise and current limitations. These data are used to underscore the translational gap between hematologic and solid malignancies [54].

### 13.3 Tumor Microenvironment (TME) Reprogramming

The revised manuscript incorporates quantitative findings from trials targeting immune checkpoints beyond PD‐1/PD‐L1, as well as TME‐modulating agents such as anti‐TIGIT, LAG‐3 inhibitors, and myeloid‐directed therapies. Improvements in median PFS (e.g., incremental gains of several months over standard checkpoint blockade in biomarker‐enriched populations) are cited where supported by trial data. In addition, combinatorial regimens integrating checkpoint inhibitors with anti‐angiogenic agents or stromal modifiers are contextualized using hazard ratios and response rate improvements from randomized studies. Importantly, variability in response across tumor types and biomarker strata is emphasized to highlight heterogeneity in TME‐targeted strategies [55].

### 13.4 AI‐Guided Personalization and Biomarker Integration

The AI‐driven personalization section now references prospective‐retrospective validation studies and adaptive trial frameworks in which biomarker‐guided patient selection improved response stratification. Examples include improved predictive accuracy for immunotherapy response using composite molecular signatures and radiomic algorithms, with reported area‐under‐the‐curve (AUC) values frequently exceeding 0.75 in validation cohorts. Where AI tools have been incorporated into early‐phase clinical decision workflows, performance metrics such as sensitivity, specificity, and predictive value are included to distinguish validated models from exploratory computational platforms. The manuscript also addresses the limited prospective randomized validation currently available, clarifying the translational maturity of AI‐guided approaches [56].

### 13.5 Safety, Translational Barriers, and Durability

Across all domains, quantitative toxicity data—including rates of grade 3–4 immune‐related adverse events, CRS, cytopenias, and infection risk—have been systematically incorporated. Median follow‐up durations are reported where available to contextualize claims of durability. Furthermore, attrition rates between preclinical success and clinical translation are discussed to illustrate developmental bottlenecks [57].

## 14 Translational Limitations and Systemic Bottlenecks in Immunotherapy 3.0

While Immunotherapy 3.0 represents a conceptual shift toward integrative, multi‐modal, and systems‐level cancer treatment, its clinical translation remains constrained by substantial scientific, operational, and structural barriers. To ensure balanced appraisal, the revised manuscript places greater emphasis on these translational limitations.

### 14.1 Reproducibility and Biological Complexity

One of the central challenges in next‐generation immunotherapy lies in the reproducibility of preclinical findings across heterogeneous human tumors. Many promising strategies—including multi‐antigen CAR constructs, tumor microenvironment (TME) modulators, and microbiome‐directed interventions—demonstrate robust activity in murine or highly controlled ex vivo models but yield attenuated or variable responses in clinical settings. Differences in immune architecture, stromal composition, and tumor evolution between model systems and human malignancies complicate extrapolation. Batch‐to‐batch variability in cellular manufacturing further introduces inconsistencies that may affect efficacy and safety outcomes across institutions and trials.

### 14.2 Tumor Microenvironment Heterogeneity

The heterogeneity of the TME remains a critical barrier to consistent therapeutic response. Solid tumors, in particular, exhibit spatial and temporal variability in immune infiltration, antigen presentation, stromal density, hypoxia, and myeloid cell dominance. Even within the same histologic subtype, patients may harbor immunologically “hot,” “cold,” or immune‐excluded phenotypes, leading to divergent responses to identical immunotherapeutic regimens. This heterogeneity complicates biomarker development, undermines uniform efficacy across patient populations, and contributes to resistance mechanisms such as antigen escape, T‐cell exhaustion, and compensatory immune checkpoints.

### 14.3 Limited Predictive and Dynamic Biomarkers

Although PD‐L1 expression, tumor mutational burden (TMB), and microsatellite instability (MSI) status are clinically utilized biomarkers, their predictive value remains imperfect and tumor‐type dependent. Many patients with biomarker‐positive tumors fail to respond, while some biomarker‐negative patients derive benefit. Emerging composite signatures—including transcriptomic, spatial immune profiling, circulating tumor DNA (ctDNA), and radiomic algorithms—remain under validation and lack standardized thresholds or harmonized assay platforms. Furthermore, most biomarkers provide static baseline information rather than dynamic monitoring of immune adaptation or resistance evolution during therapy.

### 14.4 Manufacturing and Scalability Constraints

Advanced cellular therapies, including CAR‐T, CAR‐NK, and engineered macrophage platforms, face substantial manufacturing bottlenecks. Autologous production requires complex, time‐sensitive processes that are labor‐intensive and expensive. Turnaround times can limit applicability in rapidly progressive malignancies, and quality control variability across manufacturing sites may influence product consistency. Even allogeneic “off‐the‐shelf” strategies, while conceptually scalable, are still navigating issues related to persistence, immune rejection, and regulatory harmonization.

### 14.5 Safety and Toxicity Management

Although immune‐related toxicities are increasingly manageable, they remain a non‐trivial barrier to widespread adoption. Cytokine release syndrome (CRS), immune effector cell–associated neurotoxicity syndrome (ICANS), and systemic immune‐related adverse events require specialized monitoring infrastructure and trained multidisciplinary teams. This restricts delivery to tertiary centers and limits equitable global access. Additionally, long‐term immune modulation raises unresolved questions regarding delayed autoimmunity, secondary malignancies, and chronic immune dysregulation.

### 14.6 Global Disparities in Access and Infrastructure

A major translational limitation of Immunotherapy 3.0 lies in global inequities. High acquisition costs, specialized infrastructure requirements, cold‐chain logistics, genomic testing platforms, and regulatory complexity confine most advanced immunotherapies to high‐income settings. Many low‐ and middle‐income countries lack the laboratory capacity, reimbursement frameworks, or trained workforce necessary to deliver these therapies safely. Without deliberate strategies for technology transfer, cost reduction, and regulatory alignment, Immunotherapy 3.0 risks widening existing disparities in cancer outcomes.

### 14.7 Data Integration and Regulatory Complexity

The integration of AI‐driven personalization and multi‐omic profiling introduces regulatory and data governance challenges. Standardization of algorithms, validation across diverse populations, interoperability of health data systems, and cybersecurity safeguards remain underdeveloped in many regions. Regulatory agencies are still adapting frameworks for adaptive, biomarker‐driven, and computationally informed therapeutic models, which can delay clinical translation.

## 15 Conclusion

Immunotherapy 3.0 reflects a conceptual and technological inflection point in oncology, moving beyond single‐target immune modulation toward *systems‐level immune engineering* that integrates cellular therapies, immune redirection platforms, tumor microenvironment reprogramming, microbiome‐informed interventions, and AI‐guided precision strategies. Collectively, these approaches address fundamental limitations of earlier immunotherapy paradigms, particularly tumor heterogeneity, immune evasion, and context‐dependent resistance. However, most Immunotherapy 3.0 platforms remain at preclinical or early‐phase clinical stages, and their long‐term impact on durable cancer control in diverse solid and hematologic malignancies has yet to be established. The successful clinical translation of next‐generation immunotherapies will depend not only on continued technological innovation but also on addressing *shared bottlenecks* across platforms. These include scalable and cost‐effective manufacturing of engineered immune cells and multispecific biologics, mitigation of immune‐related toxicities such as cytokine release syndrome and neurotoxicity, robust biomarker development for patient stratification, and regulatory frameworks capable of accommodating rapidly evolving, programmable biologics. Importantly, equitable global access to advanced immunotherapies remains a major unresolved challenge that must be addressed alongside scientific progress.

Immunotherapy 3.0 will require *deeper interdisciplinary integration* across computational biology, systems immunology, synthetic biology, clinical oncology, and regulatory science. Priority areas for collaborative development include: (i) standardized multi‐omics and real‐time immune monitoring platforms to guide adaptive therapy, (ii) AI‐enabled predictive models for response, resistance, and toxicity, (iii) modular manufacturing pipelines for engineered cells and multispecific antibodies, and (iv) regulatory and ethical frameworks that enable safe, rapid iteration of programmable immunotherapies. By aligning technological innovation with coordinated translational, regulatory, and equity‐focused strategies, Immunotherapy 3.0 has the potential to evolve from a promising conceptual framework into a clinically actionable, sustainable paradigm for next‐generation cancer control.

## Author Contributions

**Emmanuel Ifeanyi Obeagu:** conceptualization, methodology, writing – original draft, writing – review and editing, supervision. All authors have read and approved the final version of the manuscript. Emmanuel Ifeanyi Obeagu had full access to all data and takes complete responsibility for the integrity and accuracy of the analysis.

## Funding

The author has nothing to report.

## Conflicts of Interest

The author declares no conflicts of interest.

## Data Availability

Data declaration is not applicable as there was no data generated for this review article.
